# Multi-trait Genomic Prediction Model Increased the Predictive Ability for Agronomic and Malting Quality Traits in Barley (*Hordeum vulgare* L.)

**DOI:** 10.1534/g3.119.400968

**Published:** 2020-01-23

**Authors:** Madhav Bhatta, Lucia Gutierrez, Lorena Cammarota, Fernanda Cardozo, Silvia Germán, Blanca Gómez-Guerrero, María Fernanda Pardo, Valeria Lanaro, Mercedes Sayas, Ariel J. Castro

**Affiliations:** * Department of Agronomy, University of Wisconsin-Madison, 1575 Linden Dr., WI, 53706,; † Department of plant production, Facultad de Agronomía, Universidad de la República, Ruta 3, Km363, Paysandú 60000, Uruguay,; ‡ Maltería Uruguay S.A. Ruta 55, Km26, Ombúes de Lavalle, Uruguay,; § Instituto Nacional de Investigación Agropuecuaria, Estación Experimental La Estanzuela, Ruta 50, Km11, Colonia, Uruguay,; ** Latitud, LATU Foundation, Av Italia 6201, Montevideo 11500, Uruguay, and; †† Maltería Oriental S.A., Camino Abrevadero 5525, Montevideo 12400, Uruguay

**Keywords:** Multi-trait, multi-environment, genomic prediction, malting quality, grain yield, grain quality, Genomic Prediction, GenPred, Shared Data Resources

## Abstract

Plant breeders regularly evaluate multiple traits across multiple environments, which opens an avenue for using multiple traits in genomic prediction models. We assessed the potential of multi-trait (MT) genomic prediction model through evaluating several strategies of incorporating multiple traits (eight agronomic and malting quality traits) into the prediction models with two cross-validation schemes (CV1, predicting new lines with genotypic information only and CV2, predicting partially phenotyped lines using both genotypic and phenotypic information from correlated traits) in barley. The predictive ability was similar for single (ST-CV1) and multi-trait (MT-CV1) models to predict new lines. However, the predictive ability for agronomic traits was considerably increased when partially phenotyped lines (MT-CV2) were used. The predictive ability for grain yield using the MT-CV2 model with other agronomic traits resulted in 57% and 61% higher predictive ability than ST-CV1 and MT-CV1 models, respectively. Therefore, complex traits such as grain yield are better predicted when correlated traits are used. Similarly, a considerable increase in the predictive ability of malting quality traits was observed when correlated traits were used. The predictive ability for grain protein content using the MT-CV2 model with both agronomic and malting traits resulted in a 76% higher predictive ability than ST-CV1 and MT-CV1 models. Additionally, the higher predictive ability for new environments was obtained for all traits using the MT-CV2 model compared to the MT-CV1 model. This study showed the potential of improving the genomic prediction of complex traits by incorporating the information from multiple traits (cost-friendly and easy to measure traits) collected throughout breeding programs which could assist in speeding up breeding cycles.

Barley is the fourth most important cereal crop worldwide with an annual production of more than 143 million tons and is mainly used for animal feed and malting (FAOSTAT, 2019). Therefore, barley breeding efforts have focused mainly on grain yield and malting quality traits (Thorwarth *et al.*, 2017). However, the collection of phenotypic data such as malting quality traits in large-scale breeding programs is expensive and time-consuming. Additionally, malting quality traits (*i.e.*, beta-glucan content, malt extract, soluble nitrogen, and protein content) are complex in nature, have quantitative inheritance, and are evaluated mainly by seed destructive methods (Han *et al.*, 1997; Igartua *et al.*, 2000; Gutiérrez *et al.*, 2011; Nielsen *et al.*, 2016). Although there is an advancement in the high-throughput phenotyping techniques, it is still expensive to evaluate complex traits such as grain yield and malting quality traits with high heritability for a large set of genotypes. On the other hand, the cost of genotyping is decreasing, allowing to genotype a large number of lines at a very low cost. Therefore, some commonly used breeding strategies to overcome the challenges of complex traits is to use molecular markers (quantitative trait loci/marker-trait associations/all genomic information) for predicting and transferring these traits (Igartua *et al.*, 2000; Schmierer *et al.*, 2005; Lorenz *et al.*, 2012; Sallam *et al.*, 2015; Gutiérrez *et al.*, 2015; Schmidt *et al.*, 2016; Nielsen *et al.*, 2016; Lado *et al.*, 2018).

The ultimate goal of plant breeding is to improve the genetic gain, which is defined as the gain in the performance of a population per year through selection. In modern plant breeding, the expected genetic gain per year is defined as Δ*G*=*i r* σ*_A_*/*t*, where Δ*G* is the response to selection, *i* is the intensity of selection, *r* is the correlation between true breeding values and genotypic values, σ*_A_* is the square root of the additive genetic variance (*i.e.*, standard deviation of the breeding value), and *t* is the length of breeding cycle (Xu *et al.*, 2017). Genomic selection is a recent approach that increases genetic gain per unit of time by accelerating the breeding process through estimating the merit of an individual with the use of all the markers from the entire genome (Meuwissen *et al.*, 2001; Resende *et al.*, 2012) but without phenotyping it. The first step in genomic selection is to build the prediction model for estimating the marker effect using a training population (*i.e.*, with phenotypic and genotypic data). Then, the model is validated through cross-validation. Finally, genotypic or genomic estimated breeding values from a breeding or selection population are predicted based on their genotypic information (Lorenz *et al.*, 2012; Lado *et al.*, 2016, 2018; Xu *et al.*, 2017) and the model. However, increasing prediction accuracies of the testing population remains an area of continuous research for quantitative/complex traits such as grain yield and quality traits (Sallam *et al.*, 2015; Lado *et al.*, 2016, 2018). The predictive ability of a genomic selection model is the correlation between the true and the predicted genetic (or breeding) value from a cross-validation scheme (Lorenz *et al.*, 2012; Lado *et al.*, 2016, 2018; Xu *et al.*, 2017). The predictive ability is affected by population size (Crossa *et al.*, 2013; Zhang *et al.*, 2016; Duangjit *et al.*, 2016; Berro *et al.*, 2019), population structure (Endelman *et al.*, 2014; Heslot *et al.*, 2015; Duangjit *et al.*, 2016; Berro *et al.*, 2019), relationship between training and testing sets (Crossa *et al.*, 2014; Duangjit *et al.*, 2016; Thorwarth *et al.*, 2017), marker density (Poland and Rutkoski 2016; Duangjit *et al.*, 2016; Thorwarth *et al.*, 2017), trait architecture, heritability (Sallam *et al.*, 2015; Duangjit *et al.*, 2016; Lozada and Carter 2019), and the statistical model (Lado *et al.*, 2016, 2018; Xu *et al.*, 2017; Lozada and Carter 2019).

Multi-trait (MT) genomic prediction models use the information from individual lines and multiple traits simultaneously. Recent studies have evaluated the impact of MT prediction in genomic prediction models. For instance, a study of 495 wheat advanced breeding lines has included multiple baking quality traits in the genomic prediction model and identified that the predictive ability of unobserved individuals was increased compared to the single-trait (ST) model (Lado *et al.*, 2018). Jia and Jannink (2012) and Jiang *et al.* (2015) have incorporated genetically correlated multiple traits in prediction models and identified a significant improvement in predictive ability compared to ST models. Montesinos-López *et al.* (2016) have found higher predictive ability when using correlated traits compared to uncorrelated traits in the MT prediction model. Similarly, several other studies have observed an increase in predictive ability when multiple traits were included in the prediction models (Rutkoski *et al.*, 2012; Guo *et al.*, 2014b; Sun *et al.*, 2017; Hayes *et al.*, 2017; Lozada and Carter 2019). The use of correlated traits could be especially valuable for predicting expensive or difficult to measure traits (Lado *et al.*, 2018) such as malting quality traits in barley. The main objectives of this study were to determine whether incorporating multiple agronomic traits into the genomic prediction models would increase the predictive ability of an agronomic trait of interest such as yield, and whether the inclusion of an agronomic trait or both agronomic and malting quality traits would increase the predictive ability of a malting quality trait of interest in barley.

## Materials and Methods

### Experimental materials

Four crosses using five parents were performed to create a large inter-connected population of 980 doubled haploid lines (INNO1, CLE268 x Kalena; INNO2, Kalena x CLE267; INNO3, Kalena x Conchita; and INNO4, Livia x CLE268) at the *Instituto Nacional de Investigacion Agropecuria* (INIA), Uruguay (Figure S1). A total of 145 doubled haploid lines from those crosses were used for the model comparisons (33 lines from INNO1, CLE268 x Kalena; 39 from INNO2, Kalena x CLE267; 45 from INNO3, Kalena x Conchita; and 28 from INNO4, Livia x CLE268, Figure S1). The 145 lines were evaluated for agronomic traits for two years (2015 and 2016) in three locations [EELE (57°42’W, 30°20’S), MOSA (58°02’W, 33°16’S in 2015 and 58°04’W, 33°19’S in 2016), and MUSA (57°58’W, 33°54’S in 2015 and 57°53’W, 34°01’S in 2016)] and three years (2015, 2016, and 2017) in another location [EEMAC (58°03’W, 32°22’S)]. This resulted in a total of nine environments (location-year combinations; EELE15, EELE16, EEMAC15, EEMAC16, EEMAC17, MOSA15, MOSA16, MUSA15, and MUSA16) for evaluating agronomic traits. Malting quality traits were evaluated from samples of three of the experiments EEMAC15, EELE15, and MUSA15. All the experiments were conducted in a partially replicated experiment (Cullis *et al.*, 2006) with eight replicated lines in each experiment augmented in a randomized complete block design. The agronomic traits measured in this study were grain yield (kg ha^-1^), number of grains per square meter, thousand grain weight (g), and grain plumpness (%; the percentage weight of grains retained over a 2.5 mm sieve). The thousand grain weight and number of grains per square meter were not measured in 2016 at MUSA. The malting quality traits evaluated were beta-glucan content (ppm), malt extract (% dry basis), soluble nitrogen (mg per 100 g), and grain protein content (%). Micro-malting was performed at the *Laboratorio Tecnológico del Uruguay* (LATU) and Malteria Oriental S.A. following a standard malting procedure.

### Phenotypic data analysis

The estimation of the genotypic best linear unbiased estimates (BLUEs) was obtained from the following model:

$$y_{ijkl}=\text{μ}+E_{i}+B_{j(i)}{}_{\;}+G_{k}+GE_{ik}{}_{\;}+e_{ijkl}{}_{\;}$$

where y*_ijkl_* is the trait of interest; μ is overall mean; E*_i_* is the effect of the i-th environment (location-year combination, location, or year); B*_j(i)_* is the effect of the j-th block nested within the i-th environment; $G_{k}$ is the effect of the k-th genotype; GE*_jk_* is the effect of the genotype by environment interaction; and e*_ijkl_* is the residual error. Genotype was assumed as a fixed effect, whereas environment, block nested within the environment, and genotype by environment interactions (GEI) were assumed as random effects. For individual experiments, BLUEs were estimated using the above-mentioned model excluding environment (E) and GEI effects. These analyses were performed using PROC MIXED in SAS 9.4 (SAS Institute 2018) using restricted maximum likelihood estimates for random effects.

Broad-sense heritability for combined experiments was calculated using the model described by Cullis *et al.* (2006):

$$H^{2}=1-\frac{{\bar{v}}_{BLUP}}{2\sigma^{2}g}$$

where, ${\bar{v}}_{BLUP}$ is the average pairwise variance of the best linear unbiased predictor (BLUP) and *σ^2^_g_* is genotypic variance estimated using the ‘sommer’ package in R (Covarrubias-Pazaran 2018).

Pearson correlations among traits and environments were estimated based on the BLUEs using the *rcorr* function of ‘Hmisc’ package in R (Harrell 2019). Principal component (PC) analysis was performed for all traits based on the correlation matrix to further understand the association between traits using the ‘factoextra’ package in R (Kassambara and Mundt 2017).

### DNA extraction and SNP genotyping

Leaf tissue samples were collected from all 980 lines and DNA was extracted using the CTAB method (Saghai-Maroof *et al.*, 1984). Genotyping was performed using the 50K single nucleotide polymorphism (SNP) iSelect array at the Cereal Crops Research, Fargo, ND. SNP alleles were called using GenomeStudio software v2.0.4 (Illumina, San Diego, California). There were two main steps involved in the SNP calling using GenomeStudio. The first step involved performing SNP calls using the default clustering algorithm implemented in GenomeStudio and determining the three distinct clusters corresponding to the AA, AB, and BB genotypes. The second step involved manual curation of SNP allele clusters that were unable to clearly be identified using the default algorithm (Guo *et al.*, 2014a). The accuracy of SNP calling was validated by looking at the cluster performed, call rate value (>0.95%), and GenTrain score (>0.24). To further improve the quality of SNPs called, SNPs with call frequency <95%, minor allele frequency <0.05 and SNPs that were unmapped on barley chromosomes were removed from the analysis. This process resulted in 6,482 high-quality polymorphic SNPs that were distributed across the seven barley chromosomes (Figure S2). For genomic prediction models, SNPs were converted to -1, 0, and +1, where -1 indicates minor allele at a given locus, 0 indicates heterozygous loci, and +1 indicates major allele at a given locus. The additive relationship matrix (*K*) was estimated using the ‘A.mat’ function in the ‘rrBLUP’ package in R (Endelman 2011).

### Genomic prediction models and cross-validation schemes

**Figure 1:**
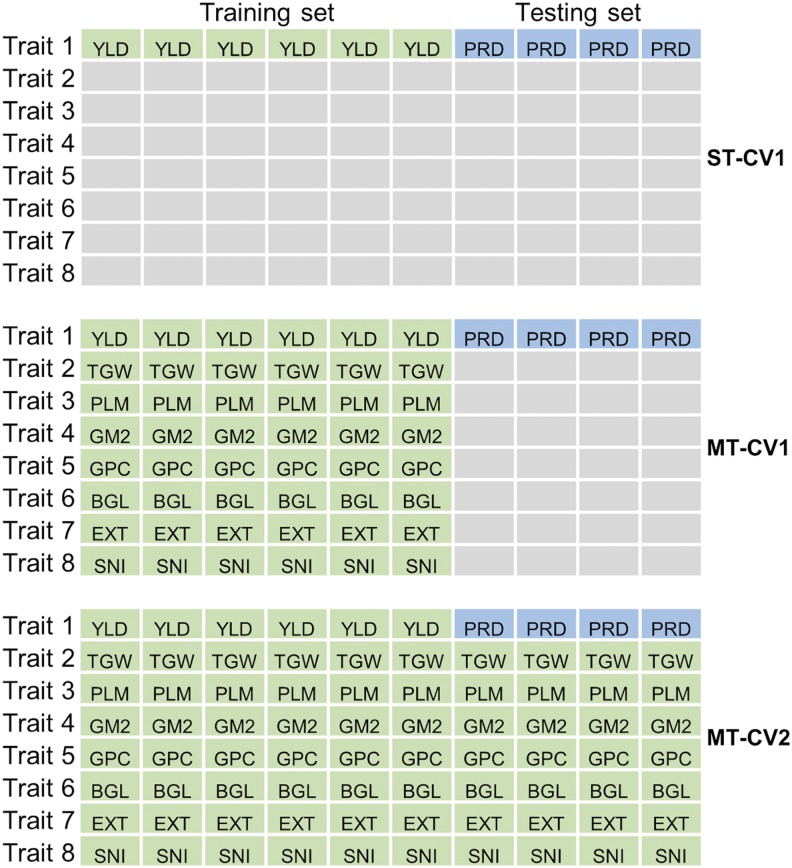
Prediction scheme for the single trait (ST) and multi-trait (MT) genomic prediction models with two cross-validation schemes (CV1 and CV2) used in this study. ST-CV1 model: single trait prediction model with cross-validation scheme 1 where a trait (*e.g.*, grain yield; YLD) is predicted at a time; we used 60% of individuals as the training set (phenotyped and genotyped, light green) and 40% of the individuals as the testing set (genotyped only, light blue with PRD code for the trait to be predicted, yield as an example here). MT-CV1 model: multi-trait prediction model with cross-validation scheme 1 for new un-phenotyped individuals; we used 60% of individuals as the training set (phenotyped for all traits and genotyped; light green) and 40% of the individuals as the testing set (genotyped but not phenotyped for any trait; light blue with PRD code for the trait to be predicted, yield as an example here). MT-CV2 model: multi-trait prediction model with cross-validation scheme 2 where 100% of the information from other traits are available for the individuals to be predicted; we used 60% of individuals as the training set (phenotyped for all traits and genotyped; light green) and 40% of the individuals as the testing set (phenotyped for associated traits but not for the targeted traits, and genotyped; light blue with PRD code for the trait to be predicted, yield as an example here). Rectangles represent genotypes and colors represent whether the phenotypic information was used (light green) or not (light blue with PRD code for the trait to be predicted, yield as an example here) for the population. Similar scheme was applied for predicting thousand grain weight (TGW), grain plumpness (PLM), number of grains per square meter (GM2), protein content (GPC), beta-glucan content (BGL), malt extract (EXT), and soluble nitrogen (SNI) where PRD was used for TGW, PLM, GM2, GPC, BGL, EXT and SNI one at a time.

Genomic prediction models were evaluated with the 145 lines that had genotypic and phenotypic information. Single trait (ST) and multi-trait (MT) models for genomic prediction of agronomic and malting quality traits were obtained following Lado *et al.* (2018). In brief, the ST model was estimated using a Bayesian ridge regression model for each trait with 1,500 burn-in and 3,000 iterations for the Gibbs sampler algorithm implemented in the ‘BGLR’ R package (Pérez and de los Campos 2014). The predictive ability was obtained for all agronomic and malting quality traits using the following ST model.

y=1μ+Zu+e

where **y** is the vector (*n* x 1) of phenotype on *n* genotypes for a single trait; **1** is a vector (*n* x 1) of ones of length *n*; **μ** is the overall mean; **Z** is the incidence matrix (*n* x *p*) with fixed known values of *p* markers for these genotypes, **u**_(_*_n_*_x1)_ is a genotypic predictor with u∼*N*(0, K*_n_*_x_*_n_*$\sigma_{g}^{2}$), where **K** is the realized additive relationship matrix and $\sigma_{g}^{2}$ is additive genetic variance; and **e** is the residual errors vector with e∼*N*(**0**, **R*_n_*_x_*_n_***$\;\sigma_{e}^{2}$) where **R** accounts for heterogeneity in mean estimate precision. The predictive abilities for the ST model were estimated using only one cross-validation scheme (CV1) as shown in Figure 1.

The MT model was built using a Bayesian multivariate Gaussian model estimating an unstructured variance-covariance matrix between traits (**Σ**) and residual matrix (**R**) with 1,500 burn-in and 3,000 iterations for the Gibbs sample algorithm implemented in the ‘MTM’ R package (de los Campos and Grüneberg 2016) as described by Lado *et al.* (2018). The model used was:

y=1μ+Zu+e

where **y** is a vector of *n* x *t* length (*n* genotypes and *t* traits); **1** is a vector of ones of length *n*x*t*; **μ** is the overall mean; **Z**_[(*n*x*t*)xp]_ is the incidence matrix; **u**_[_*_n_*_x_*_t_*_)x1]_ is a genotypic predictor with **u** ∼ MVN(0, **Σ**⊗**K**) and **e** is the residual errors vector with **e** ∼ MVN(**0**, **R**⊗**I**), where **Σ** is the variance-covariance matrix across traits, **K** is the realized additive relationship matrix among individuals estimated from the markers, **R** is the variance-covariance matrix for the residual effects for each individual in all traits, and I is the *n*x*n* identify matrix. **Σ** was estimated as an unstructured matrix and **R** as a diagonal matrix following Lado *et al.* (2018). The predictive abilities for the MT model were estimated using two cross-validation schemes (CV1 and CV2) as described in Figure 1.

Two genomic prediction models (ST and MT) were used to compare two main cross-validation schemes described previously (Lado *et al.*, 2018) (Figure 1). Briefly, the first cross-validation scheme (CV1) used a random set of lines (60%; ∼90) with phenotypic and genotypic information (training set) to train the model and the remaining lines (testing set; 40%; ∼60) for prediction using genotypic information only. This process was repeated 100 times, where each iteration included different lines in the training and testing sets. The second cross-validation scheme (CV2) used a random set of lines (60%) with phenotypic and genotypic information for the trait of interest and other multiple traits (*i.e.*, correlated traits) to train the model. The remaining lines (40%) were predicted for the trait of interest using genotypic and phenotypic information from other correlated traits. This process was repeated 100 times where each iteration included different lines in the training and testing sets. The first cross-validation scheme was used for the ST (designated as ST-CV1) and MT (MT-CV1) models, whereas the second cross-validation scheme was used only for the MT (MT-CV2) model. In short, the CV2 scheme uses full genotypic information on every individual (training and prediction sets), full phenotypic information for correlated traits on every individual (training and prediction sets), and phenotypic information of trait of interest only on the training set. Therefore, CV2 only applies to multiple traits problems. Pearson’s correlation (*r_PA_*) between the genotypic values and genotypic BLUEs was estimated to determine the predictive ability of the models.

Genomic prediction models for agronomic traits were tested using the BLUEs from an individual experiment, experiments across locations (EELE, EEMAC, MOSA, and MUSA), years (2015 and 2016), and all combined (designated as ‘ALL’). Similarly, genomic prediction models for malting quality traits were tested using BLUEs from individual experiments conducted in 2015 (EELE15, EEMAC15, MOSA15, and MUSA15), experiments combined across locations in 2015 (‘ALL15’) and using agronomic trait information from all nine experiments combined (ALL). Agronomic traits used in the genomic prediction models were grain yield, thousand grain weight, number of grains per square meter, and grain plumpness. For the MT model, two strategies were used for predicting agronomic traits of interest. The first strategy included predicting the agronomic trait of interest using all other three agronomic traits (AGRO). The second strategy included predicting the agronomic trait of interest using the remaining three agronomic traits and all four malting quality traits (A+M). Similarly, five strategies were used for predicting malting quality trait of interest as follows: (i) all four agronomic traits (AGRO), (ii) both agronomic and malting traits (A+M), (iii) positively correlated traits with grain protein content (COR1; beta-glucan content, grain plumpness, soluble nitrogen, and grain protein content), and (iv) traits explaining the largest variance in the first principal component (PC1 or COR2: grain yield, thousand grain weight, number of grains per square meter, and grain protein content); and (v) traits explaining the largest variance on the second principal component (PC2 or COR3: beta-glucan content, grain plumpness, soluble nitrogen, and malt extract).

### Data availability

File S1 contains supplementary Tables S1-S6 and Figures S1-S6. Table S1 contains basic summary statistics with BLUEs, standard error, minimum, maximum, coefficient of variation, broad sense heritability, and proportion of variance component for genotype by the environment and genotypic effect for agronomic and malting quality traits evaluated across multiple locations and years. Table S2 contains an analysis of variance with mean squares for agronomic and malting quality traits. Table S3 contains genomic predictive ability for un-phenotyped environments using MT-CV1 and MT-CV2 models. Table S4-S6 contains predictive ability for agronomic and malting quality traits using MT-CV1 and MT-CV2 models. Figure S1 shows a principal component analysis of all individuals. Figure S2 contains a distribution of the 6,482 single nucleotide polymorphisms (SNPs) across the seven chromosomes of barley in 145 doubled haploid lines. Figures S3-S6 contain Pearson’s correlation among environments (locations-years) for grain yield, number of grains per square meter, grain plumpness, and thousand grain weight, respectively. File S2 contains the BLUEs for the agronomic traits (grain yield, plumpness, thousand grain weight, and number of grains per square meter) from individual experiments (EELE15, EELE16, EEMAC15, EEMAC16, EEMAC17, MOSA15, MOSA16, MUSA15, and MUSA16), experiments across locations (EELE, EEMAC, MOSA, and MUSA), years (2015, 2016, and 2017), and all experiments combined (ALL). File S3 contains the BLUEs for the malting quality traits (beta-glucan content, malt extract, protein content, and soluble nitrogen) from the experiments conducted in 2015 in three locations (EELE, EEMAC, and MOSA) and for the combined experiments (ALL15). Supplemental material available at figshare: https://doi.org/10.25387/g3.9172619.

## Results

### Multi-trait characterization

Basic summary statistics and analysis of variance of the agronomic and malting quality traits are in Tables 1, S1, and S2. A wide range of variation was observed for agronomic and malting quality traits across environments (EELE, EEMAC, MOSA, and MUSA). The highest mean grain yield averaged over years was observed at MOSA (7,658 kg ha^-1^), followed by EELE (7,337 kg ha^-1^), EEMAC (5,134 kg ha^-1^), and MUSA (3,877 kg ha^-1^). Similarly, a wide variation across environments was observed for other traits.

**Table 1:** Best linear unbiased predictors (BLUEs) and standard deviation (in parenthesis) for each environment (*i.e.*, combination of location-year) evaluated for agronomic and malting quality traits in in Uruguay

	Agronomic				Malting Quality			
Location	Grain yield (kg ha^-1^)	Thousand grain weight (g)	No. of grains per m^2^	Grain plumpness (%)	Malt extract (%)	Beta-glucan content (ppm)	Soluble Nitrogen (mg per 100g)	Grain protein content
——————————————————————— 2015 ———————————————————————								
EELE	9131 (849.2)	50.5 (2.5)	18062 (1860.4)	97.8 (0.9)	81.2 (1.0)	158.1 (89.7)	618.2 (62.6)	10.6 (0.7)
EEMAC	5406 (918.4)	44.1 (3.0)	12314 (2206.7)	88.5 (4.7)	81.0 (0.9)	387.0 (156.0)	595.2 (51.1)	10.9 (0.7)
MOSA	7134 (1034.4)	41.1 (3.8)	17466 (2742.2)	82.4 (8.3)	80.2 (1.1)	259.2 (111.0)	676.2 (59.6)	10.8 (0.7)
MUSA	3801 (854.4)	40.3 (3.7)	9640 (2384.0)	87.3 (5.5)	—	—	—	—
——————————————————————— 2016 ———————————————————————								
EELE	5535 (883.3)	45.6 (2.2)	12257 (2125.3)	94.8 (1.7)	—	—	—	—
EEMAC	4934 (588.7)	47.4 (2.8)	10500 (1448.0)	97.3 (1.5)	—	—	—	—
MOSA	8192 (741.4)	50.3 (3.8)	16326 (2047.2)	96.7 (1.9)	—	—	—	—
MUSA	3937 (1034.5)	94.7 (2.0)	—	—	—	—	—	—
——————————————————————— 2017 ———————————————————————								
EEMAC	5163 (1374.9)	37.9 (5.7)	13741 (3630.4)	66.5 (14.1)	—	—	—	—

Variance component analysis showed that the proportion of the variance explained by the GEI effects to genotypic effects was greater than one for most of the agronomic traits whereas lower than one for malting quality traits (Table S1). Broad-sense heritability estimates for agronomic traits were low to high ranging from 0.20 to 0.80 (Table S1). Low to moderate heritability was observed for grain yield (0.20 to 0.66) and plumpness (0.25 to 0.53) whereas moderate to high heritability was observed for number of grains per square meter (0.46 to 0.71) and thousand grain weight (0.42 to 0.80). Relatively high heritability was observed for malting quality traits ranging from 0.66 (beta-glucan content) to 0.74 (soluble nitrogen) (Table S1).

**Figure 2:**
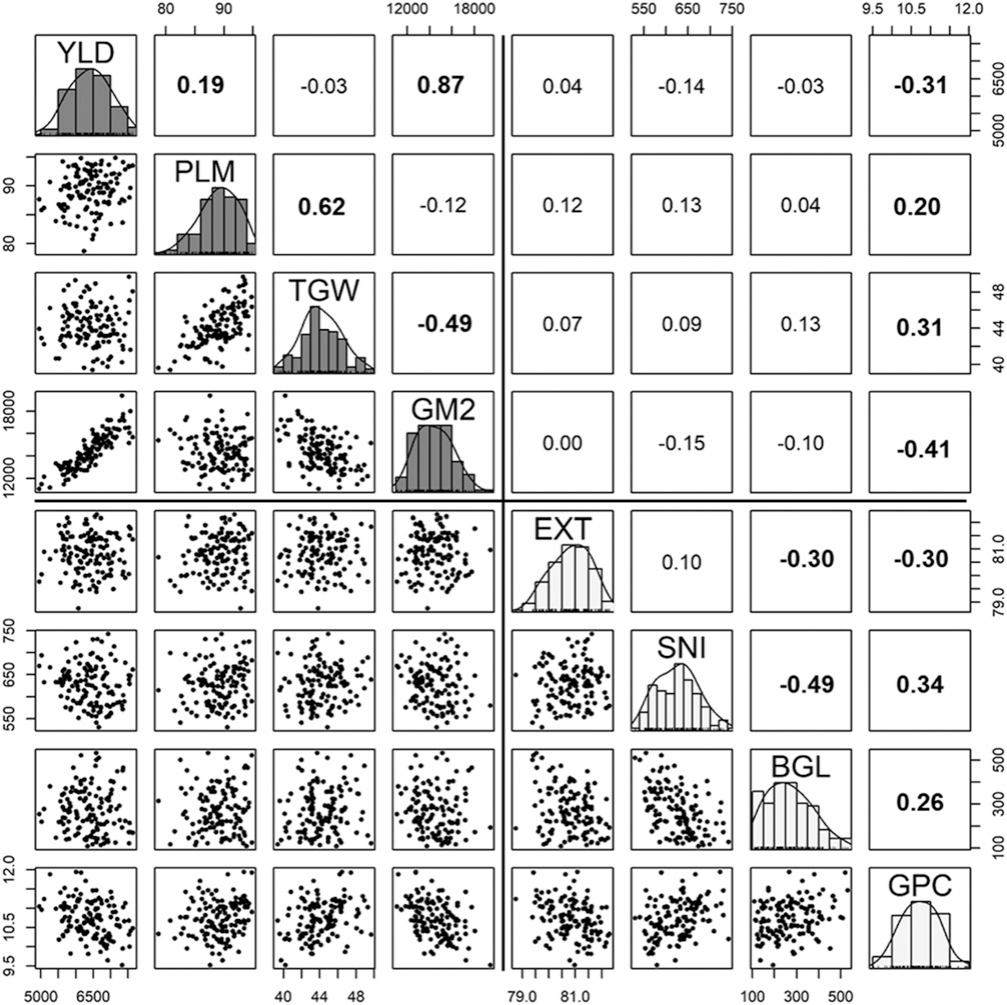
Genetic correlation between agronomic and malting quality traits using best linear unbiased estimates from combining three experiments (EEMAC, EELE, and MOSA) conducted in 2015. Numbers in bold indicate a correlation significantly different from zero at an alpha level of 0.05. BGL, beta-glucan content; EXT, malt extract; GM2, number of grain m^-2^; PLM, grain plumpness; TGW, thousand grain weight; GPC, grain protein content; SNI, soluble nitrogen; and YLD, grain yield.

Grain yield was positively correlated with plumpness and the number of grains per square meter, negatively correlated with grain protein content, and not correlated with grain weight (Figure 2). Grain protein content affects the malting quality of barley as indicated by the positive correlation with beta-glucan content, soluble nitrogen, and grain plumpness, and negative correlation with malt extract. Additionally, grain protein content was correlated with both yield components (positive correlation with grain weight and negative correlation with the number of grains). The correlation values for all agronomic traits were mostly higher among locations than years (Figures S3-S6).

**Figure 3:**
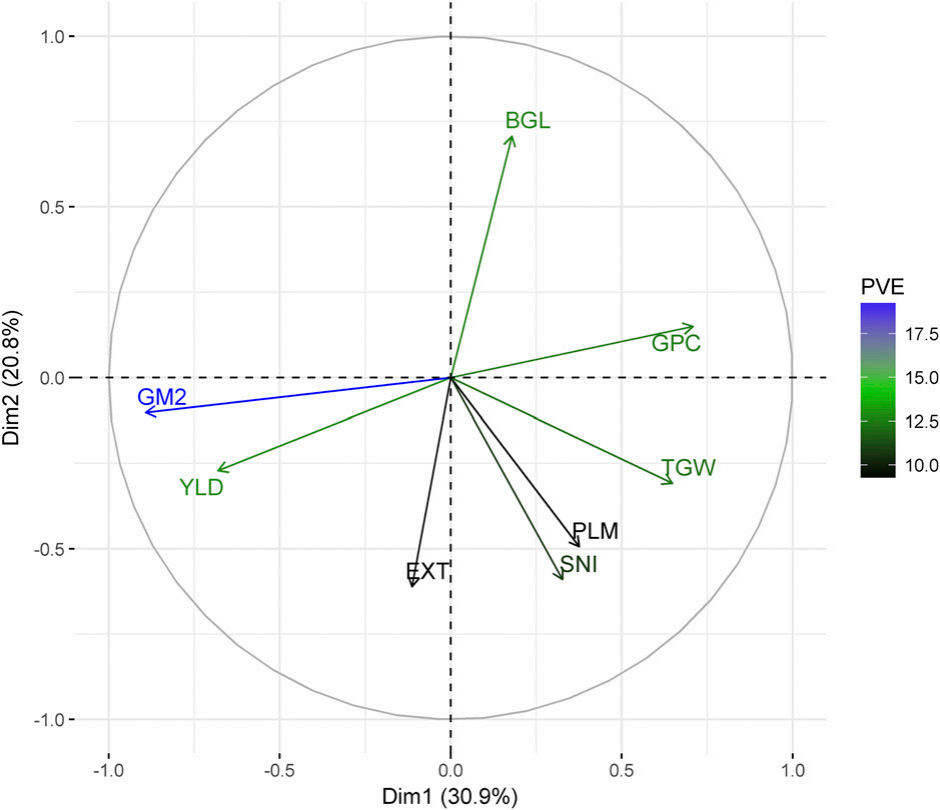
Principal component analysis showing the association between agronomic and malting quality traits. BGL, beta-glucan content; EXT, malt extract; GM2, number of grains per square meter; PLM, grain plumpness; TGW, thousand grain weight; GPC, grain protein content; SNI, soluble nitrogen; and YLD, grain yield. PVE: Proportion of the variance explained by each trait.

In the principal component analysis (PCA), the first two PCs explained ∼52% of the total variation (Figure 3). The PC1 was mostly explained by yield component traits, such as grain yield, protein content, number of grains per square meter, and thousand grain weight. The PC2 was mostly explained by malting quality traits such as beta-glucan content, malt extract, grain plumpness, and soluble nitrogen.

### Genomic prediction for agronomic traits

**Figure 4:**
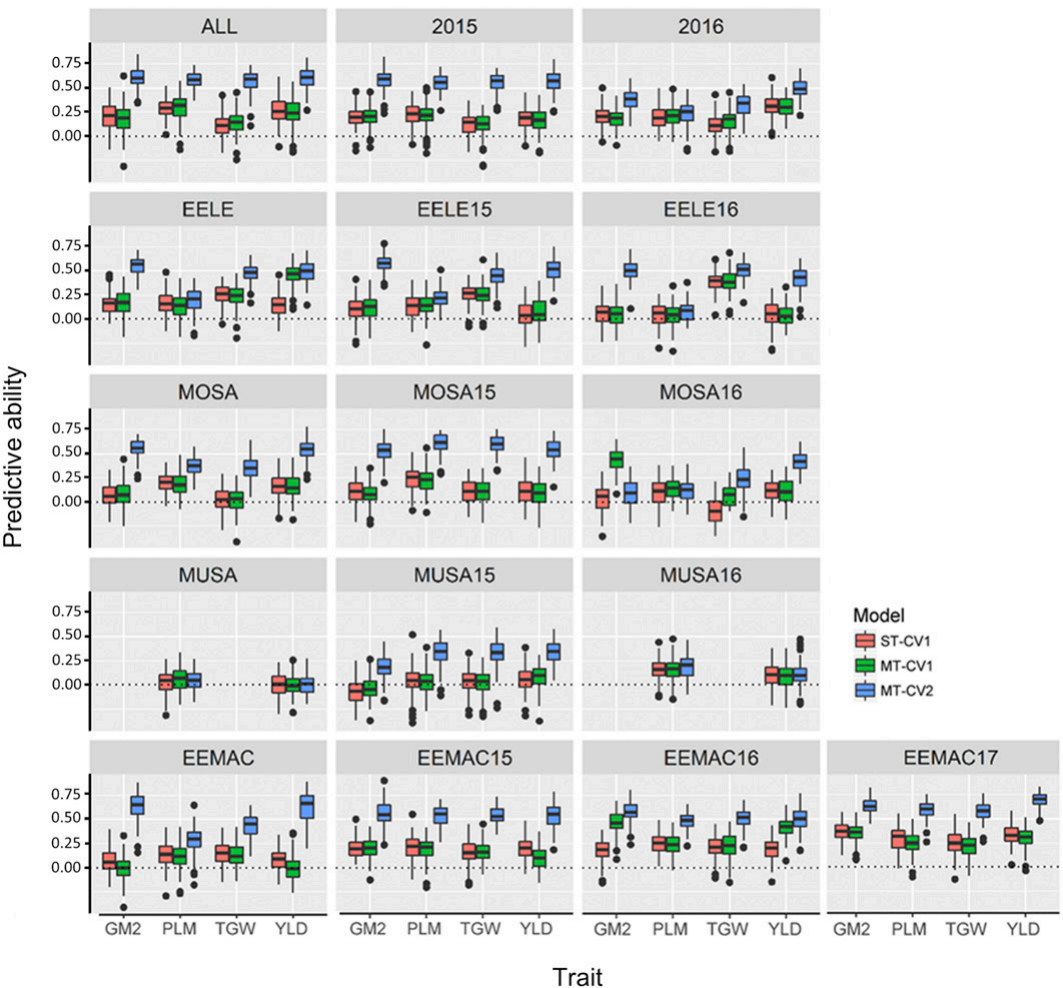
Box plots showing the predictive ability for grain yield (YLD), plumpness (PLM), thousand grain weight (TGW), and number of grains per square meter (GM2) using single trait (ST) and multi-trait (MT) models from individual experiments (EELE15, EELE16, EEMAC15, EEMAC16, EEMAC17, MOSA15, MOSA16, MUSA15, and MUSA16), experiments across locations (EELE, EEMAC, MOSA, and MUSA), years (2015, 2016, and 2017), and all experiments combined (ALL). CV1, predicting new lines with genotypic information only and CV2, predicting partially phenotyped lines by using genotypic and phenotypic information from all traits from individuals in the training set, and genotypic and correlated phenotypic traits in the testing set.

The model (MT-CV2) that included other agronomic traits from individuals to be predicted performed better for agronomic traits than either single (ST-CV1) or multi-trait (MT-CV1) models without this information (Figure 4 and Table S4). For instance, the predictive ability for grain yield using the MT-CV2 model was high ranging from 0.34 to 0.70 for most environments. Low predictive ability was found for MUSA16 and MUSA combined. The MUSA16 environment, where agronomic traits were weakly correlated, was an outlier. The predictive ability was high for all agronomic traits ranging from 0.56 to 0.60 using the MT-CV2 model when all experiments were combined (Figure 4 and Table S4).

**Figure 5:**
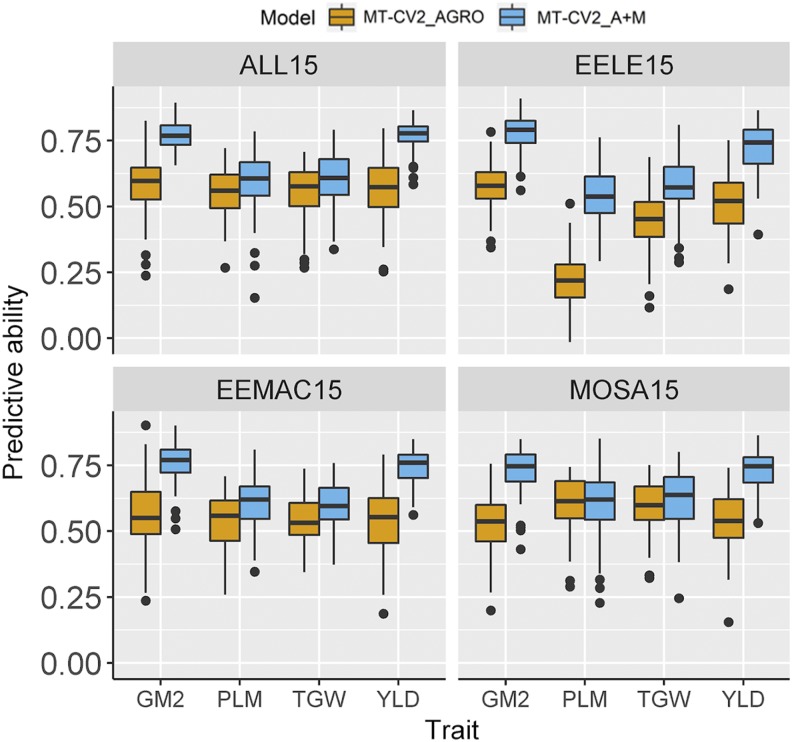
Boxplots showing the predictive ability for grain yield (YLD), plumpness (PLM), thousand grain weight (TGW), and number of grains square meter (GM2) using single trait (ST) and multi-trait (MT) models from 2015 experiments conducted in three locations (EELE, EEMAC, and MOSA). ALL15, three combined experiments of the year 2015; CV2, predicting partially phenotyped lines by using genotypic and phenotypic information from all traits from individuals in the training set, and genotypic and phenotypic information from other correlated traits in the testing set.; The correlated traits were: AGRO, agronomic traits (YLD, PLM, TGW, and GM2); or A+M, agronomic and malting quality traits (beta-glucan content, malt extract, protein content, and soluble nitrogen).

To identify whether the inclusion of additional traits such as malting quality traits along with the agronomic traits in an MT model could further improve the predictive performance of agronomic traits of interest, we evaluated a multi-trait (MT-CV2) prediction model including both agronomic and malting (A+M) traits and compared it with the MT model including agronomic (AGRO) traits only (Figure 5 and Table S5). The predictive ability for the agronomic trait of interest was further increased from using both A+M traits compared to the model with AGRO traits only. For instance, the mean predictive ability of the MT-CV2 model at all combined experiments in 2015 for grain yield using only agronomic traits was 0.57 compared to 0.77 when using both agronomic and malting traits.

### Genomic prediction for malting quality traits

**Figure 6:**
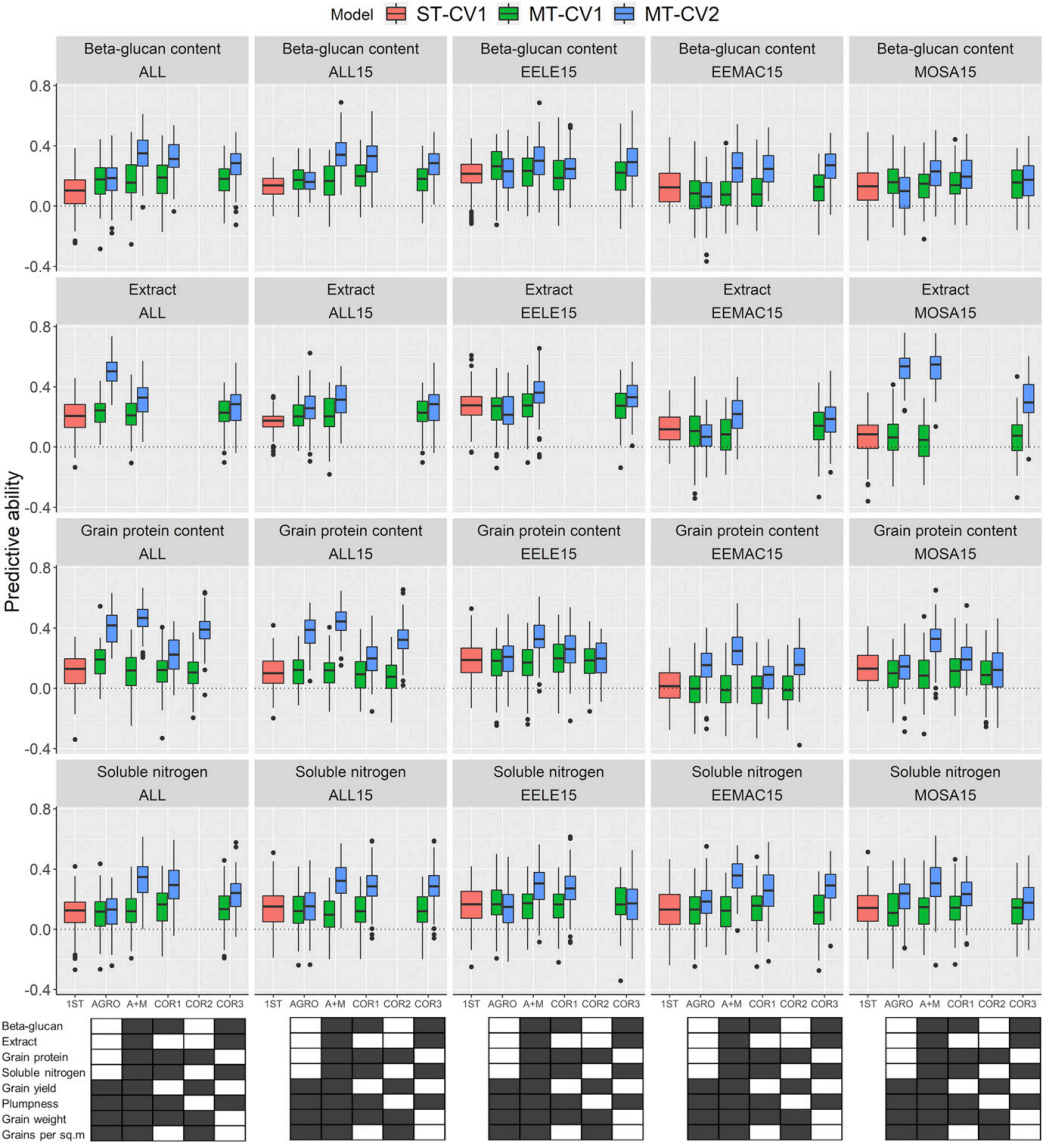
Box plots showing the predictive ability for malting quality traits using single trait (ST) and multi-trait (MT) prediction models for the experiments conducted in 2015 in three locations (EELE, EEMAC, and MOSA) and for the combined experiments in 2015 (ALL15) and all (ALL). ALL, agronomic traits from all nine experiments combined and malting traits from the ALL15; CV1, predicting new lines with genotypic information only; and CV2, predicting partially phenotyped lines by using genotypic and phenotypic information from all traits from individuals in the training set, and genotypic and phenotypic information from other correlated traits in the testing set. Predicted traits were: beta-glucan content; malt extract; protein content; and soluble nitrogen; ST represents single trait, AGRO, agronomic traits (grain yield, plumpness, thousand grain weight, and number of grains per square meter) and trait of interest for multi-trait prediction model; A+M, agronomic and malting quality traits; COR1, denotes correlated traits which are soluble nitrogen, thousand grain weight, beta-glucan content, plumpness, and grain protein content; COR2, denotes traits within principal component 1 (PC1), which are grain yield, thousand grain weight, number of grains per square meter, and grain protein content; COR3, traits within PC2 which are beta-glucan content, plumpness, soluble nitrogen, and extract. Treatment combinations were shown in a 5 × 8 rectangle figure where each small rectangle with colors represent the presence (black) or absence (white) of phenotypic information used in the prediction model.

A substantial increase in the predictive ability of the malting quality trait of interest was observed using the MT-CV2 model containing A+M and other correlated traits (COR1, COR2, and COR3) in the prediction model for both individual and combined experiments (Figure 6 and Table S6). Additionally, the predictive ability of grain protein content was increased using the MT-CV2 model containing only agronomic traits. However, the predictive ability of ST-CV1, MT-CV1, and MT-CV2 models was similar when we used only the agronomic traits to predict other malting quality traits. The predictive ability of the MT-CV2 model that uses both agronomic and malting quality traits had the highest predictive ability for malting quality traits followed by the models that included correlated traits. For instance, the predictive abilities for grain protein content using MT-CV2 models with A+M traits ranged from 0.25 to 0.46. Similarly, the predictive abilities for grain protein content using MT-CV2 that included correlated traits ranged from 0.07 to 0.38. The grain protein predictive ability at all combined experiments using the MT-CV2 model with A+M had 76.2% and 75.8% higher predictive ability than using the MT-CV1 model with A+M and ST-CV1 models, respectively.

### Genomic prediction in un-phenotyped environment

Genomic prediction for a location using the information from other years (*e.g.*, predict 2017 from 2015 and 2016 information and vice versa) had relatively lower predictive ability compared to the prediction for a location using information from other locations (*e.g.*, predict EELE from using EEMAC, MOSA, and MUSA) (Table S3). In general, genomic prediction using the MT-CV2 model had a higher predictive ability for all traits compared to the MT-CV1 model for unobserved environments. For instance, grain yield predictive ability across locations using the MT-CV1 was up to 0.26 and the MT-CV2 model was up to 0.41. For prediction across years, the MT-CV1 model was up to 0.27 and the MT-CV2 was up to 0.32. The highest predictive abilities using MT-CV2 for grain plumpness, thousand grain weight, and number of grains per square meter were 0.52, 0.53, 0.52, and 0.46, respectively. For locations, on average, the highest predictive ability was observed at EEMAC for grain yield, MOSA for plumpness, EELE for thousand grain weight, and MUSA for number of grains per sq. m using the MT-CV2 model.

## Discussion

Plant breeders routinely collect multiple traits for evaluating the performance of genotypes in multiple environments. The selection of genotypes based on genome-wide markers and phenotypic information using genomic selection methods is gaining popularity in breeding programs with the advent of cost-effective and robust next-generation sequencing technologies (Poland and Rife 2012). However, only a few studies have been conducted using multi-trait multi-environment genomic selection methods due to complexity of model and requirements of high computing power (Calus and Veerkamp 2011; Jia and Jannink 2012; Rutkoski *et al.*, 2015; Lado *et al.*, 2018; Ward *et al.*, 2019). The present study investigated the different strategies for including multiple traits in multi-environment trials for predicting un-phenotyped or partially phenotyped lines.

### Trait characterization and association

The first thing to do before utilizing multiple traits in a genomic prediction model is to understand the characteristics of the traits used. This study evaluated four agronomic traits (grain yield, thousand grain weight, grain plumpness, and number of grains per square meters) and four malting quality traits (beta-glucan content, malt extract, protein content, and soluble nitrogen) for use in a multi-trait genomic selection model. The proportion of variance explained by GEI was larger for agronomic traits than malting traits (Table S1), suggesting a large influence of GEI effects on agronomic traits compared to quality traits. The larger influence of GEI on agronomic traits resulted in a lower heritability for agronomic traits compared to quality traits. Lower heritability estimates for agronomic traits such as grain yield was expected as they are governed by many genes and are genetically complex in nature (Thorwarth *et al.*, 2017; Bhatta *et al.*, 2018b). Similar results were observed for agronomic and quality traits in previous studies (Rode *et al.*, 2012; Sallam *et al.*, 2015; Thorwarth *et al.*, 2017).

For the MT model for agronomic traits, two sets of traits (set 1: grain yield and number of grains per square meter; and set 2: plumpness and thousand grain weight) were strongly positively correlated to each other. The positive correlation of grain yield with number of grains per square meter and no correlation with grain weight in the current study indicates that the grain yield was largely defined by the number of grains per square meter component. The negative relationship between grain yield and protein content identified in this study is well understood in cereal crops and is mainly associated with the dilution effect (Bertholdsson 1999; Bhatta *et al.*, 2018a). Similarly, the positive association of thousand grain weight and a negative association of malt extract with grain protein content have been reported previously in malting barley cultivars (Bertholdsson 1999; Dai *et al.*, 2007).

### Multi-trait genomic prediction models and their implications in a breeding program

Increasing genomic predictive ability of quantitative and complex traits such as grain yield and malting quality traits are primary goals for successful utilization of genomic selections in breeding programs (Sallam *et al.*, 2015; Lado *et al.*, 2016, 2018). In this study, we have evaluated eight traits with different range of heritabilities and correlations to investigate strategies for integrating multiple traits into the prediction model. The main aim of the study was to determine whether incorporating multiple agronomic traits or both agronomic and malting quality traits into the multi-trait models (MT-CV1 and MT-CV2) could further improve the predictive ability of a trait of interest compared to single trait (ST-CV1) model. The present study suggests little to no advantage of the MT-CV1 model, irrespective of different multiple traits inclusion strategies, compared to the ST-CV1 model when predicting new genotypes. Newly introduced genotypes are untested for multiple traits across environments and using the MT-CV1 under this scenario may not provide additional information to leverage for increasing predictive ability (Burgueño *et al.*, 2012; Ward *et al.*, 2019). Also, the predictive ability using MT-CV1 and ST-CV1 was low for grain yield (0.07 to 0.17 for MT-CV1 and 0.05 to 0.15 for ST-CV1). Similar results were observed in previous studies where the MT-CV1 model that included correlated traits did not perform better than the ST-CV1 model (Calus and Veerkamp 2011; Schulthess *et al.*, 2016; Dos Santos *et al.*, 2016; Lado *et al.*, 2018), demonstrating that the multi-trait models are not necessarily advantageous for new individuals compared to the single-trait model. This result may be associated with the trait complexity and wide range of heritability of the multiple traits used (Jia and Jannink 2012; Lado *et al.*, 2018). However, a few studies have identified considerable increase in predictive ability due to the inclusion of correlated traits with high heritability in MT-CV1 models (Rutkoski *et al.*, 2012; Jia and Jannink 2012; Guo *et al.*, 2014b; Montesinos-López *et al.*, 2016), but the benefit of MT-CV1 model for complex trait is minimal compared to the ST-CV1 model (Jia and Jannink 2012).

The MT-CV1 and ST-CV1 models did not show differences, it is, therefore, similar to compare ST-CV1 to MT-CV2. The MT-CV2 model for all studied traits significantly improved the predictive ability for the trait of interest compared to MT-CV1 and ST-CV1 models. Additionally, the MT-CV2 model had higher predictive abilities for all agronomic traits (0.56 to 0.60) across all combined experiments. For instance, the MT-CV2 model for grain yield across all combined experiments increased the predictive ability by 57% and 61% compared to the ST-CV1 and MT-CV1 models, respectively. This may be associated with the higher heritability estimates of combined environments compared to individual environments. The higher predictive ability in genomic prediction models for a trait is expected when the heritability is higher (Jia and Jannink 2012; Guo *et al.*, 2014b; Thorwarth *et al.*, 2017; Wang *et al.* 2017). This result suggested that the MT-CV2 model can provide fairly high genomic predictive performance for a complex trait such as grain yield for multi-environment trials when high heritability and correlated traits are used. Similarly, Wang *et al.* (2017) have also reported a higher predictive ability for grain yield when using the multi-trait model in rice. A genomic prediction study in 750 winter barley genotypes identified low predictive ability for grain yield (0.31) and high predictive ability for thousand grain weight (0.71) (Thorwarth *et al.*, 2017) comparable to the predictive abilities of the MT-CV2 model in our study. This result suggested that the MT-CV2 model could be useful in predicting traits after partially phenotyping complex traits such as grain yield. Furthermore, the predictive ability of agronomic trait of interest using the MT-CV2 model was improved using both agronomic and malting traits compared to using agronomic traits only. For example, the MT-CV2 model with both agronomic and malting traits for grain yield across all combined experiments in 2015 increased predictive ability by 26% compared to the MT-CV2 model with agronomic traits only. Evaluating malting quality traits for predicting the agronomic traits may not have a practical implication on a breeding program, however, the most interesting aspect is that if researchers are evaluating malting quality traits for their study, using this information will improve the yield prediction. This result showed the potential strategy for further improving complex traits such as grain yield via the inclusion of a larger number of highly heritable and correlated traits. On the other hand, the predictive ability of the MT-CV2 model that included weakly correlated traits such as grain yield and plumpness at MUSA and MUSA16 had similar predictive ability compared to MT-CV1 and ST-CV1 models. This result was expected due to the fact that uncorrelated/weakly correlated traits have rarely useful information from the correlation matrix that goes into the multi-trait prediction model (Jia and Jannink 2012; Montesinos-López *et al.*, 2016; Wang *et al.*, 2017; Lado *et al.* 2018), thereby, no additional improvement in the predictive ability was observed.

Malting quality traits are expensive, time-consuming, labor-intensive, and difficult to breed due to their complex genetic architecture (Gutiérrez *et al.*, 2011). Nevertheless, in malting barley production, they are generally the key and final factor determining if an experimental line can be developed into a commercial variety. Using information from correlated traits could increase the predictive performance of the trait of interest (Jia and Jannink 2012; Wang *et al.*, 2017). In this study, the highest predictive ability for malting quality traits was observed when using the MT-CV2 model including both agronomic and malting quality traits followed by the model including some correlated traits. These results suggested that the prediction of quality traits in genomic selection could be improved by borrowing information from correlated agronomic traits. A recent study on multi-trait genomic prediction in bread baking quality in 495 advanced wheat lines has reported increased predictive ability from the MT-CV2 model that included one highly correlated trait compared to MT-CV1 and ST-CV1 models (Lado *et al.*, 2018). Several other studies have also reported advantages of the multi-trait prediction model for predicting traits of partially phenotyped individuals (Rutkoski *et al.*, 2012; Jia and Jannink 2012; Guo *et al.*, 2014b; Sun *et al.*, 2017; Hayes *et al.*, 2017). This result suggested that the MT-CV2 model that included correlated traits should be used as a strategic approach to replace phenotyping of labor-intensive and high-cost traits such as malting traits. However, the predictive abilities of ST-CV1, MT-CV1, and MT-CV2 models were similar when we used only the agronomic traits that were uncorrelated with malting quality traits to predict malting quality trait of interest.

Additionally, this study showed that the predictive ability using the MT-CV2 model could be further increased with the increase in the number of associated traits included in the prediction model. Similarly, a previous study on 575 rice hybrids had reported an increase in the predictive ability with the increase in the number of multiple traits included in the prediction model (Wang *et al.*, 2017). However, other studies have shown that the contributions of multiple traits in the model would tend asymptotically toward zero, and additional traits could introduce issues in co-linearity; and therefore, fewer traits have been recommended (Schulthess *et al.*, 2016; Lado *et al.*, 2018; Lozada and Carter 2019).

Similarly, genomic prediction in un-phenotyped environments using the MT-CV2 model outperformed the MT-CV1 model. This result suggests that the MT-CV2 model should be used for predicting the performance of genotypes in new environments when phenotyping was performed for other traits. On average, predicting a location using other location data resulted in a higher prediction compared to predicting across the year. This result may be associated with higher correlations among locations compared to correlations among years where high GEI was observed. Wang *et al.* (2017) had observed a higher predictive ability of the MT multi-environment model compared to the ST single environment model because MT multi-traits models can borrow information from associated traits across environments. This study showed the usefulness of the MT-CV2 model in predicting a new environment despite the presence of GEI.

However, there are several factors affecting the predictive abilities of MT multi-environment genomic prediction models. Some of the important factors associated with this study were the size and composition of the training population (Crossa *et al.*, 2013; Zhang *et al.*, 2016; Duangjit *et al.*, 2016; Berro *et al.*, 2019), the genetic relationship between the training and testing population (Crossa *et al.*, 2014; Duangjit *et al.*, 2016; Thorwarth *et al.*, 2017), trait genetic architecture and heritability (Sallam *et al.*, 2015; Duangjit *et al.*, 2016; Wang *et al.*, 2017; Lozada and Carter 2019), trait correlations (Lozada and Carter 2019), correlations among environments (Lado *et al.*, 2016), and GEI (Heslot *et al.*, 2014; Lado *et al.*, 2016). Although the population size in our study was small, the predictive ability using correlated traits in the CV2 scheme had high PA, suggesting that correlated traits could somewhat offset the effect of smaller population sizes.

Although poor predictive ability remains a major challenge in implementing genomic selection (Crossa *et al.*, 2014), several studies have shown that genomic selection could be advantageous for complex traits with low heritability such as grain yield (Belamkar *et al.*, 2018; Burgueño *et al.*, 2012; Lozada *et al.*, 2019). Genomic selection with low to moderate levels of predictive ability could be used in early-generation testing and selection (Belamkar *et al.*, 2018; Juliana *et al.*, 2018; Michel *et al.*, 2018) and in off-season nurseries where field phenotypic information might be useless. Breeding program do not rely on predictive ability itself, but also on how genomic selection can be leveraged in selecting or discarding lines within a program, selecting parents, and phenotyping efforts (Belamkar *et al.*, 2018; Juliana *et al.*, 2018; Lado *et al.*, 2018).

## Conclusions

Multiple traits such as agronomic and malting quality traits are routinely evaluated in barley breeding programs. In general, every experimental line goes through an extensive selection process which includes a high number of experiments, all of them including agronomic phenotyping and a fraction of them including malting quality testing (due to cost and logistical reasons). Genomic selection using multiple traits collected throughout the breeding program could be useful for improving the genomic prediction of complex traits of interest. We evaluated strategies for adding multiple traits and compared their impact on the predictive ability of the single (ST-CV1) and multi-trait genomic prediction models (MT-CV1 and MT-CV2) on the predicted genotypic values for a trait of interest in barley breeding program. We found that including agronomic traits that are relatively easy to score and inexpensive improves the prediction of more complex traits such as grain yield and expensive/difficult to measure traits such as malting quality traits. These results can have a large impact on breeding programs where improving grain yield is a major goal and malting quality evaluations are not performed routinely in all individuals, but agronomic evaluations are. Our results suggest that including genotypic information and a model that includes all the collected data, breeders can improve the identification of superior lines with a small increase in the costs. Using these strategies could significantly change the way that phenotyping trials are set up in the future, and also the way that individuals are chosen to be phenotyped for malting quality traits.
